# Effects of mefloquine and artesunate mefloquine on the emergence, clearance and sex ratio of *Plasmodium falciparum *gametocytes in malarious children

**DOI:** 10.1186/1475-2875-8-297

**Published:** 2009-12-16

**Authors:** Akintunde Sowunmi, Oluchi O Nkogho, Titilope M Okuboyejo, Grace O Gbotosho, Christian T Happi, Elsie O Adewoye

**Affiliations:** 1 Department of Pharmacology & Therapeutics, Institute for Medical Research and Training, University College Hospital, Ibadan, Nigeria; 2 Department of Physiology, University of Ibadan, Ibadan, Nigeria

## Abstract

**Background:**

The gametocyte sex ratio of *Plasmodium falciparum*, defined as the proportion of gametocytes that are male, may influence transmission but little is known of the effects of mefloquine or artesunate-mefloquine on gametocyte sex ratio and on the sex ratio of first appearing gametocytes.

**Methods:**

350 children with uncomplicated *P. falciparum *malaria were enrolled in prospective treatment trial of mefloquine or artesunate-mefloquine between 2007 and 2008. Gametocytaemia was quantified, and gametocytes were sexed by morphological appearance, before and following treatment. The area under curve of gametocyte density *versus *time (AUC_gm_) was calculated by linear trapezoidal method.

**Results:**

91% and 96% of all gametocytes appeared by day 7 and day 14, respectively following treatment. The overall rate of gametocytaemia with both treatments was 31%, and was significantly higher in mefloquine than in artesunate-mefloquine treated children if no gametocyte was present a day after treatment began (25.3% *v *12.8%, P = 0.01). Gametocyte clearance was significantly faster with artesunate-mefloquine (1.8 ± 0.22 [sem] *v *5.6 ± 0.95 d; P = 0.001). AUC_gm _was significantly lower in the artesunate mefloquine group (P = 0.008). The pre-treatment sex ratio was male-biased, but post-treatment sex ratio or the sex ratio of first appearing gametocytes, was significantly lower and female-biased two or three days after beginning of treatment in children given artesunate-mefloquine.

**Conclusion:**

Addition of artesunate to mefloquine significantly modified the emergence, clearance, and densities of gametocytes and has short-lived, but significant, sex ratio modifying effects in children from this endemic area.

## Background

Gametocytes are crucial for malaria transmission from the human host to the mosquito vector. Although the exact mechanisms of gametocytogenesis are unclear and many individual mosquitoes that ingest gametocytes do not support their development to sporozoite stage [1], anti-malarials, as well as other factors such as host genetic or immune status may significantly influence the process of gametocytogenesis and the development of gametocytes in individual mosquitoes [2,3].

The persistence, in peripheral blood, of *Plasmodiun falciparum *gametocytes has been estimated to vary from 3-21 days [4-6]. However, the emergence and clearance gametocytes have been less frequently evaluated in therapeutic efficacy studies in general and have not been investigated in African children treated with mefloquine or atresunate-mefloquine in particular.

In natural populations, the *Plasmodium spp *gametocyte sex ratio is female biased, but this ratio can vary significantly during the course of individual infections [7-9]. Although anti-malarials may significantly influence the temporal changes in gametocyte sex ratio [10,11], the exact contribution of chemotherapy to these changes is difficult to quantify because many confounding variables such as gametocyte density, host anaemia, host cell preference and, kin discrimination in malaria parasites influence sex allocation strategies in the parasite [7,9,12,13].

Artesunate-mefloquine combination is increasingly used on the African continent for the treatment of acute falciparum malaria. However, the individual components of artesunate-mefloquine are readily available and used in Africa; it is desirable to evaluate the effects of both mefloquine and artesunate mefloquine on gametocyte carriage and sex ratio. In addition, it is important to assess how these drugs influence the first appearance of gametocytes in African children following treatment of uncomplicated infections. The information from these evaluations may assist in planning control measures.

The aims of the present study are to evaluate the emergence and clearance of gametocytes; the rate of gametocytaemia; the temporal changes in sex ratio; and the sex ratio of first appearing gametocytes in children treated for acute falciparum malaria with mefloquine or artesunate plus mefloquine in an endemic area of southwestern Nigeria.

## Methods

### Patients

The study was conducted between July 2007 and August 2008 in children aged ten years or below with acute uncomplicated *P. falciparum *malaria in Ibadan, southwestern Nigeria, an endemic area of malaria [14]. Fully-informed consent was obtained from the parents/guardians of each child. Inclusion criteria were: fever or history of fever in the 24-48 h preceding presentation, pure *P. falciparum *parasitaemia ≥ 2000 asexual forms/μL, a body (axillary) temperature > 37.4°C or history of fever in the 24-48 h preceding presentation, absence of other concomitant illness, no history of anti-malarial use in the two weeks preceding presentation, and negative urine tests for anti-malarial drugs (Dill-Glazko and lignin). Patients with severe malaria [15], severe malnutrition, serious underlying diseases (renal, cardiac, or hepatic), and known allergy to study drugs were excluded from the study. The study protocol was approved by the local ethics committee.

### Drug management

After clinical assessment, blood was obtained for haematocrit determination and for quantification of asexual and sexual parasitaemia. Patients were randomized to (i) a single dose regimen of mefloquine 25 mg/kg on presentation (day 0), or (ii) artesunate at 4 mg/kg daily for three days (day 0-2) plus mefloquine given as in (i) above. All drugs were given orally and all patients waited for at least three hours after to ensure the drug was not vomited. If it was, the patient was excluded form the study.

Oral paracetamol (acetaminophen) at 10-15 mg/kg 6-8 hourly was given for 12-24 h if body temperature was > 38°C. Patients were seen daily, at approximately the same time of the day for the first eight days (days 0-7) and then on days 14, 21, 28, 35 and 42 after treatment had begun. At each visit, patients were assessed clinically and thick and thin blood smears were obtained for quantification of parasitaemia. The fever clearance time was defined as the time taken for body temperature to fall to below 37.5°C and remain below this value for > 48 h.

### Laboratory investigations

Thick and thin blood films prepared from a finger prick were stained with 10% Giemsa for 30 minutes and were examined by light microscopy under an oil-immersion objective, at 1000× magnification by two independent assessors who did not know the drug treatment of the patient. A senior member of the study team reviewed the slides, if there was any disagreement between the microscopists. In addition, the slides of every third child enrolled in the study were reviewed by this senior member.

Asexual parasite and gametocyte counts were measured daily for the first eight days (days 0-7) and thereafter on days 14, 21 and 28. Quantification of asexual and sexual parasites in Giemsa-stained thick blood films was done against 500 leukocytes in the case of asexual parasitaemia, and against 1,000 leukocytes in the case of gametocytes. From this figure, the parasite density was calculated assuming a leukocyte count of 6,000/μl of blood. Asexual parasite clearance time was the time interval from the start of anti-malarial treatment until the asexual parasite count fell below detectable levels in a peripheral blood smear. Gametocyte clearance time was the interval between the first and last positive smears for gametocytes. Capillary blood, collected before and during follow-up, was used to measure packed cell volume (PCV). PCVs were measured using a microhaematocrit tube and microcentrifuge (Hawksley, Lancing, UK). Routine haematocrit was done on days 0, 3, 7, 14, 21 and 28.

### Determination of gametocyte sex and gametocyte sex ratio

Gametocyte sex determination was based on the following criteria [16,17]: males (microgametocytes) are smaller than females (macrogametocytes), the nucleus is larger in males than females, the ends of the cells are rounder in males and angular in females, with Giemsa the cytoplasm stains purple in males and deep blue in females, and the granules of malaria pigment are centrally located females and more widely scattered in males. Gametocytes were sexed if at least three of the five criteria stated above were present and if the gametocyte density was ≥ 15/μl blood. The sex ratio was defined as the proportion of gametocytes that were male [18].

For each patient, gametocyte densities were plotted against time. The areas under the curve of gametocytaemia *versus *time (AUC_gm_) were determined by a non-compartmental method using the computer programme *Turbo Ken *(Clinical Pharmacology Group, University of Southampton, UK, through the courtesy of Prof. A.G. Renwick) as previously described [11]. Briefly, AUC_gm _was obtained, using the linear trapezoidal rule from time zero (0 h, day 0) to the time of gametocyte clearance, or if there was no clearance, until 1008 h (day 42). The final gametocyte density at the time of clearance was assumed to be 0.001 sexual forms/μl blood (a level assumed to be below microscopic detection).

### Data analysis

Data were analysed using Epi-Info version 6 [19], and the statistical programme SPSS for Windows version 10.01 [20]. Variables considered in the analysis were related to the densities of *P. falciparum *gametocytes and trophozoites. Proportions were compared by calculating χ^2 ^with Yates' correction or by Fisher exact or by Mantel Haenszel tests. Normally distributed, continuous data (e.g. fever, asexual parasite and gametocyte clearance times) were compared by Student's t-tests and analysis of variance (ANOVA). Data not conforming to a normal distribution (e.g. asexual and sexual parasite densities, gametocyte sex ratio, and area under curve of gametocytaemia versus time) were compared by the Mann-Whitney U-tests and the Kruskal-Wallis tests (or by Wilcoxon ranked sum test). Kaplan-Meier plots are also presented to compare gametocyte carriage rates following treatment. Differences in survival time were assessed by inspection of Kaplan-Meier curves and log-rank tests. All tests of significance were two-tailed. P-values of < 0.05 were taken to indicate significant differences. Data were (double)-entered serially using the patients codes and were only analysed at the end of the study.

## Results

### Patients

Three hundred and fifty patients were recruited: 174 in atresunate-mefloquine group and 176 in mefloquine group. All children completed at least 14-21 days of follow up. The baseline characteristics of children with gametocytaemia are summarized in Table 1. These characteristics were similar in the two treatment groups. However, parasite but not fever clearance was significantly faster in children treated with artesunate-mefloquine.

**Table 1:** Demographic data and immediate therapeutic response of children with *P falciparum *gametocytaemia treated with mefloquine or artesunate-mefloquine

	Values* for the following treatment groups			P. value
Parameter	Artesunate-Mefloquine	Mefloquine	All	
No. of patients	44	64	108	-

Male/Female	22/22	32/32	54/54	-

Age (year)	6.1 ± 3.1	6.9 ± 2.8	6.5 ± 2.9	0.18
	1-13	0.8-13	0.8-13	
No. < 5 years	14	12	26	

Weight (kg)	18.2 ± 7.1	19.4 ± 5.6	18.9 ± 6.3	0.31
	8-46	7-33	7-46	

Height (cm)	109.2 ± 19.4	115.2 ± 16.1	112.7 ± 17.7	0.09
	65-141	74-147	65-147	

Duration of illness (d)	2.5 ± 0.9	3.1 ± 1.4	2.9 ± 1.3	0.01
	1-5	1-7	1-7	

Body temperature (°C)	38.3 ± 1.2	38.2 ± 1.2	38.2 ± 1.2	0.6
	36.2-40.5	35.2-40.5	35.2-40.8	

Parasite count (/μL)				
Geometric mean	29280	27760	28590	0.9
Range	2200-884571	2400-520000	2200-884571	

Gametocyte density (/μL)				
Geometric mean	18	28	23	0.24
Range	6-216	6-468	6-468	

Fever clearance time (d)				
	1.3 ± 0.6 (n = 30)	1.3 ± 0.8 (n = 40)	1.3 ± 0.7	0.86
	1-3	1-5	1-5	

Parasite clearance time (d)				
	1.4 ± 0.5	1.9 ± 0.8	1.7 ± 0.8	0.003
	1-3	1-4	1-4	

Data are shown as mean ± SD or median (range),

### First detection of gametocytaemia

Table 2 summarizes the times of detection of gametocytes. Overall, 72%, 91% and 96% of all gametocytes were detectable by days 3, 7, and 14, respectively. The rates of detection were similar in the two treatment groups.

**Table 2:** First appearance of gametocytes in children with *P. falciparum *malaria treated with treated with mefloquine or artesunate-mefloquine

	No. with gametocytes			
Days after enrolment	Artesunate-Mefloquine	Mefloquine	All	Cumulative %
0	22	20	42	38.9
1	3	6	9	47.2
2	6	13	19	64.8
3	3	5	8	72.2
4	1	3	4	75.9
5	1	2	3	78.7
6	1	3	4	82.4
7	3	6	9	90.7
14	3	3	6	96.3
21	-	2	2	98.1
28	-	1	1	99.1
35	1	-	1	100
42	-	-	-	100
Total	44	64	108	100

### Gametocytaemia

Gametocytes were detected in peripheral blood in 108 children (31%) from the two treatment groups. Thus the overall proportions of children with gametocytaemia was 31%. The proportion was 12.8% and 25.3% for artesunate-mefloquine and mefloquine alone, respectively if no gametocytes were present by day 1 after enrolment (Table 2). The difference between these proportions was significant (P = 0.01).

The overall detection rate at enrolment was 12% and was not significantly different between the two treatment groups: 20 of 171 (11.4%) versus 22 of 171 (12.6%), in the mefloquine and artesunate-mefloquine groups, respectively, P = 0.71. After treatment, the emergence of gametocytes was significantly less frequent in the artesunate-mefloquine group than in the mefloquine alone group: 22 of 171 (12.6%) versus 44 of 171 (25%), respectively, P = 0.004. Post-treatment, gametocyte carriage was significantly higher than pre-treatment in the mefloquine alone group (20 of 171 versus 44 of 171, P = 0.0009) but not in the artesunate-mefloquine group (22 of 171 versus 22 of 171, P = 1.00).

### Duration of gametocyte carriage

**Figure 1:**
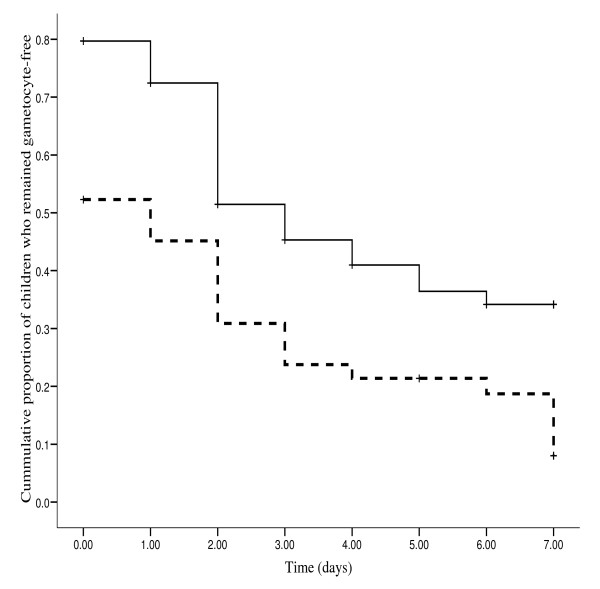
**Survival curve of cumulative probability of remaining gametocyte-free 7 days following treatment with mefloquine (solid line) or artesunate plus mefloquine (solid broken line) for gametocytaemic children (log-rank statistic = 9.85, P = 0.002)**.

The probability of mosquito infectivity is related to gametocyte density and the duration of carriage by the host. Figure 1, a Kaplan-Meier plot of the cumulative probability of being gametocyte free during the first week of follow-up was significantly higher in the artesunate-mefloquine treated than in mefloquine alone treated children (log rank statistic = 9.85, P = 0.002).

**Figure 2:**
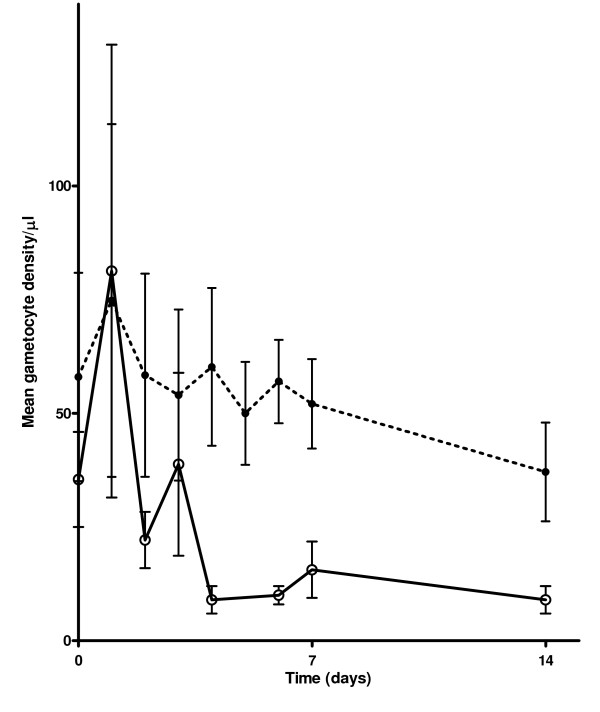
**Area under curve of gametocyte density *versus *time in children treated with mefloquine (black closed circle on dashed line) or artesunate mefloquine (open circle on solid line)**.

**Figure 3:**
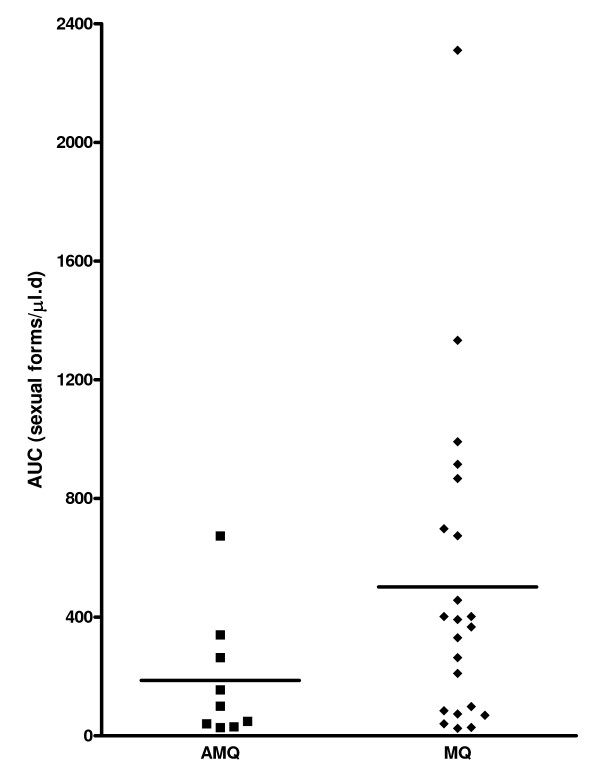
**Distribution of AUC_gm _values in children treated with mefloquine (MQ) or aresunate mefloquine (AMQ)**. Horizontal bars indicate mean values.

AUC_gm _was determined in patients who carried gametocytes at least on three occasions within 14 days. The mean AUC_gm _were (501.7 ± 115.6 (SEM), 95% confidence interval (95% CI) 261.4-742.0 sexual forms/μl.d in mefloquine-treated children and (186.7 ± 71.2 (SEM), 95% CI 22.4-351.0 sexual forms/μl.d in artesunate-mefloquine treated children (Figures 2 and 3). The difference between these values was significant (P = 0.008). The mean AUC_gm _was 2.7-fold higher in mefloquine than in artesunate-mefloquine treated children.

### Clearance of gametocytes

Only seven children, all treated with mefloquine alone, had gametocytes on or after day 14. Overall, gametocyte clearance was significantly faster in the artesunate-mefloquine group than in the mefloquine alone group (1.8 ± 0.22 [sem], range 1-8 *versus *5.6 ± 0.95, range 1-35 d; P = 0.001) (Table 3). Gametocytes were cleared within four days in 95% and 63% of children treated with artesunate-mefloquine and mefloquine alone, respectively. The difference between these proportions was significant (χ^2 ^= 13.74, P = 0.0002).

**Table 3:** Clearance of gametocyte following treatment of *P. falciparum *infections in children with mefloquine or artesunate-mefloquine

	Drug treatment		
	
Time to clearance of gametocyte (days)	Artesunate-mefloquine	Mefloquine	P. value
1	31	29	0.017

2	3	7	

3	4	3	

4	4	1	

5		2	

6	1	3	

7			

8	1	10	0.026*

11		1	

14		1	

>14		7	

GCT			
Mean ± sem	1.8 ± 0.22	5.6 ± 0.95	0.001
Range	1-8	1-35	

*Fisher exact test

### Temporal changes in sex ratio following treatment

In 64 children who had gametocytaemia at presentation or during follow-up in the mefloquine group, a total of 202, 222, 263, 163, 165, 116, 123, 176, 65, 14, 62, 95 and 26 were counted but 184, 205, 230, 134, 145, 103, 121, 165, 65, 14, 62, 95, and 26 could be sexed on days 0,1,2,3,4,5,6,7,14, 21, 28, 35 and 42, respectively. In 44 children who had gametocytaemia at presentation or during follow-up, in the artesunate plus mefloquine group, a total of 126, 122, 37, 72, 3, 3, 5, 13, 5, 3 and 2 were counted but 120, 110, 23, 65, 3, 3, 5, 11, 6, 3, and 2 could be sexed on days 0, 1, 2,3, 4, 5, 6, 7, 14, 21 and 35, respectively. Thus, very few gametocytes were present in those treated with artesunate plus mefloquine when compared with those treated with mefloquine alone.

**Figure 4:**
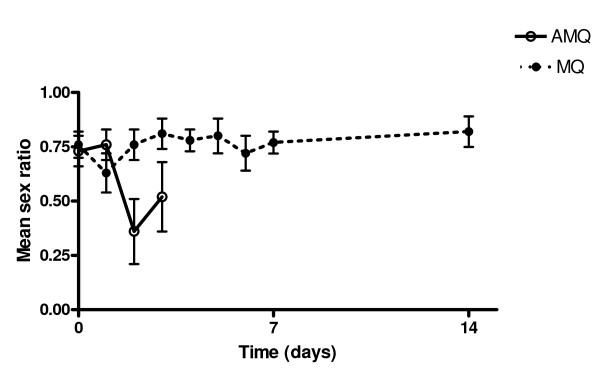
**Temporal changes in mean gametocyte sex ratio in children treated with mefloquine (MQ) or artesunate-mefloquine (AMQ)**.

The variations in sex ratio following treatment are shown in Figure 4. Sex ratio at enrolment in both treatment groups was male-biased and was not significantly different in the mefloquine and mefloquine-artesunate treated children (0.76 ± 0.07 (SEM) *v *0.73 ± 0.07, P = 0.76). However, sex ratio values 2 d after treatment began were significantly lower and more female-biased in the artesunate-mefloquine group compared with the mefloquine alone group (0.35 ± 0.15 (SEM) *v *0.76 ± 0.07, P = 0.02). In addition, this value for artesunate-mefloquine was significantly lower than pre-treatment mean sex ratio (P = 0.04). In mefloquine treated children, the corresponding values were similar (P = 0.93).

### Sex ratio of first-appearing gametocytes

**Figure 5:**
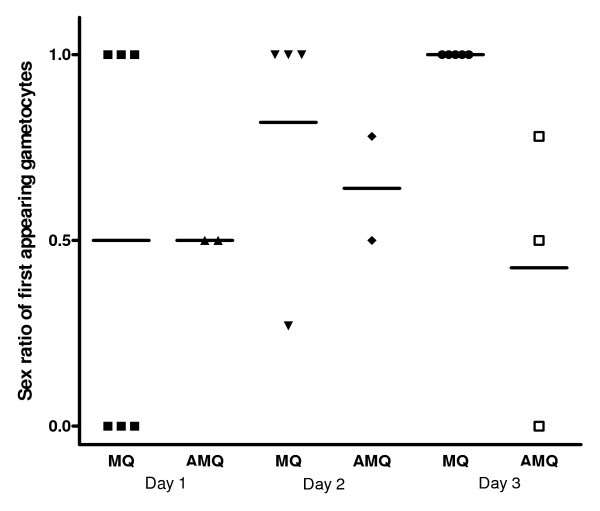
**Sex ratio of first appearing gametocytes in children treated with mefloquine (MQ) or artesunate-mefloquine (AMQ)**.

Overall, the sex ratio of first appearing gametocytes was male-biased in mefloquine treated children. In artesunate-mefloquine treated children, sex ratio of first appearing gametocytes three days after treatment began became female-biased and was significantly lower (P = 0.04) in the artesunate-mefloquine than in the mefloquine alone group (Figure 5).

## Discussion

The overall gametocyte carriage rate of 12% at enrolment is consistent with previous findings in this and other endemic areas [6,11,21-23] but may be an underestimate since sub-microscopic gametocytaemia detectable by polymerase chain reaction (PCR) is not uncommon in children from this and other endemic areas [24-26]. Indeed submicroscopic gametocytaemia is present in 15% of children from this endemic area following treatment with artemether-lumefantrine [26].

Only 40% of gametocytaemic patients were identified on presentation. This proportion is significantly lower than 72% recorded in an area of lesser intensity of transmission in Thailand [27]. The reasons for this region- or transmission intensity-related difference are not immediately apparent from the results of the present study. That 35% of all gametocytes emerged after completion of treatment (Table 2) may have implications for the present treatment policy in Africa and suggests that artemisinins and artemisinin-based combination therapy are not rapidly gametocytocidal during the early acute phase of the infections [26].

The risk of gametocyte carriage following treatment with anti-malarials varies considerably; the risk is often higher with monotherapy than with artemisinin combination therapy [6,11,22,28], and in the study area, is highest with antifolate monotherapy [11]. In the present study, the proportion of patients developing a gametocytaemia if none was present by one day after treatment began was twice as high with mefloquine compared with artesunate-mefloquine. This time-related increase in proportion of patients with gametocytaemia after administration of two daily doses of an artemisinin-based combination therapy may allow an objective assessment of the effects of anti-malarial drugs on gametocyte carriage. The time-related increase in proportion is dependent primarily on the rapidity of clearance of asexual parasitaemia with a consequently reduced chance of development of gametocytaemia and, secondarily on the rate of clearance of sexual parasitaemia. Thus, as shown in Thailand [28], the significantly rapid clearance of asexual parasitaemia by artesunate-mefloquine compared with mefloquine alone contributed to this relatively reduced proportion of patients with gametocytaemia in the present study. However, the relatively high proportion of children with gametocytaemia (12.8%) after artesunate-mefloquine, again suggests that despite relatively rapid asexual parasiticidal action, artemisinins are not gametocytocidal for mature gametocytes. In addition, this proportion is considerably greater than 1.9% recorded in an area of lesser intensity of transmission in Thailand [27], suggesting that the chance of being gametocytaemic increases as transmission intensity increases.

The significantly higher post-treatment gametocyte carriage in children treated with mefloquine alone suggest that the slow clearance of asexual parasitaemia (independent of recrudescence) may be primarily responsible for the progression of committed asexual parasites to develop to gametocytes. Recrudescence in both mefloquine and artesunate-mefloquine is associated with gametocytaemia [28,29]. The similar pre- and post-treatment gametocytaemia in those treated with artesunate-mefloquine suggests moderate effects of artesunate on gametocyte carriage. This observation, in the context of artemisinin based comnination therapy (ACT) supports a recent finding from East and West Africa [24,26].

The study evaluated the effects of treatment with mefloquine or artesunate-mefloquine on gametocyte clearance and sex ratio in children who were gametocytaemic before, during or after treatment commenced. To our knowledge, this is the first study of the effects of these drugs on gametocyte clearance and sex ratio in African children. In these children, artesunate-mefloquine cleared gametocytamia significantly more rapidly than mefloquine alone [28-30] and may prevent transmission of the infection. Although this impact may not be readily obvious in areas of intense transmission, in areas of low transmission, this may have significant impact on malaria incidence [28,31]. Compared to relatively non-immune individuals living in areas of lesser intensity of transmission in Thailand, gametocyte clearance in the cohort of children treated with artesunate-mefloquine was relatively shorter (10 days versus five days). This, in addition to other possibilities, raises a question: does immunity increase drug-assisted gametocyte clearance? This is likely since in endemic population immunity enhances the clearance of asexual parasites [32].

Monocytes and leukocytes phagocytose early and late stages of gametocytes, respectively [33,34]. It is unclear how non-gametocytocidal anti-malarials interact with this mechanism to produce clearance of gametocytes. Perhaps artemisinin drugs enhance the clearance of late gametocytes while being at the same time directly gametocytocidal to early gametocytes.

Gametocyte sex ratio may be influenced by a number of factors including anaemia, the number of and the competition between different parasite genotypes and other biological variables such as parasite density and transmission rates [7-9,12,13]. Overall, sex ratio at presentation in the cohort of children was male-biased in contrast to previous findings from this endemic area [11] suggesting that, sex ratios in natural population although female-biased may be variable [7,35]. In order to examine the effects of anti-malarials on the sex ratio allocation strategies of *P. falciparum*, an evaluation of the sex ratio of first-appearing gametocytes was made. The assumption was that the sex ratio of first-appearing gametocytes as opposed to sex ratio of all gametocyte present in circulation would be a more sensitive indicator of the effects of chemotherapy. Based on this assumption, the finding was that, overall, the sex ratio of first appearing gametocytes was male-biased except on the third day after treatment began in the artesunate-mefloquine group, when it became significantly female-biased. Since the gametocytes emerging immediately following treatment were already allocated to sex several days before treatment began, the female-biased sex ratio observed with artesunate-mefloquine would suggest that the drug has no effect on sex allocation strategy of the parasite. Therefore, it is likely that the observed female-biased sex ratio is due to preferential release, into peripheral blood, of female gametocytes or a selective killing effect on male gametocytes. The effect (s) on emergence, however, was short-lived, and may have been blunted by the relatively long half-life of mefloquine of 16-33 days [36] exerting a more profound effect than artesunate with a very short half-life. Thus, it is possible that partner drugs in artemisinin-based combinations may modify their female-biased sex ratio producing effects as previously demonstrated for artesunate-amodiaquine combination [37]. Another possible reason is that male gametocytes are longer-lived [38,39]. Studies are now needed to assess the effects of artemisinin monotherapy on the sex ratio of first appearing gametocytes and the temporal changes in the sex ratio of cohorts of first-appearing gametocytes in falciparum infections.

Plasmodium species may increase their chances of transmission by investing in gametocyte production or in sex allocation [9]. It would appear from the results of the present study that after treatment with mefloquine or artesunate-mefloquine, *P. falciparum *increased its investment significantly in gametocyte production but not in sex allocation suggesting that induction of gametocytogenesis and sex determination may have different mechanisms.

In addition to the density of peripheral gametocytes [40] and the duration of carriage, the sex ratio of the gametocyte may play a role in transmission success since the ratio may impact on mosquito infectivity [17]. Following treatment AUC_gm _was approximately three-fold higher in mefloquine than in artesunate-mefloquine treated children. Assuming sex ratio is related to mosquito infectivity, the higher value, coupled with a higher AUC_gm _treatment for mefloquine should presumably put patients treated with mefloquine at a higher risk for mosquito infectivity and transmissibility if the gametocytes were viable.

There are potential implications of the findings of the present study: primaquine is gametocytocidal and can shorten gametocyte clearance time [29,41] but its inclusion as part of artemisinin-based combination therapy is fraught with the danger of inducing haemolysis in a population where the child prevalence of glucose phosphate dehydrogenase (G6PD) deficiency may be close to 20% or where anti-malarial (dapsone)-induced haemolysis may be a potential public health problem (Premji et al., unpublished observation) or where malaria-associated anaemia is a public health problem [42] and is associated with an increased risk of gametocyte carriage and sex ratio changes that may influence transmission [43]. There is need to find combination drugs that are safe and can rapidly clear gametocytaemia in children (and adults) with falciparum malaria living in areas of intense transmission in Africa. Tafenoquine in combination with artemisinin-based combination therapy may be able to play this role.

## Competing interests

The authors declare that they have no competing interests.

## Authors' contributions

AS led the design, conduct, data analysis and manuscript preparation. OON and TMO were involved with conduct and data analysis. GOG and CTH were involved in design, conduct, and preparation of the manuscript. EOA was involved with data analysis and manuscript preparation. All authors read and approved the manuscript.
